# c-Fos-dependent miR-22 targets MDC1 and regulates DNA repair in terminally differentiated cells

**DOI:** 10.18632/oncotarget.18389

**Published:** 2017-06-07

**Authors:** Jung-Hee Lee, Seon-Joo Park, Seok Won Kim, Gurusamy Hariharasudhan, Sung-Mi Jung, Semo Jun, Jeongsik Yong, Ho Jin You

**Affiliations:** ^1^ Laboratory of Genomic Instability and Cancer Therapeutics, Cancer Mutation Research Center, Chosun University School of Medicine, Seosuk-dong, Gwangju, Republic of Korea; ^2^ Department of Cellular and Molecular Medicine, Chosun University School of Medicine, Seosuk-dong, Gwangju, Republic of Korea; ^3^ Department of Premedical Sciences, Chosun University School of Medicine, Seosuk-dong, Gwangju, Republic of Korea; ^4^ Department of Neurosurgery, Chosun University School of Medicine, Seosuk-dong, Gwangju, Republic of Korea; ^5^ Department of Pharmacology, Chosun University School of Medicine, Seosuk-dong, Gwangju, Republic of Korea; ^6^ Department of Biochemistry, Molecular Biology and Biophysics, University of Minnesota Twin Cities, Minneapolis, Minnesota, United States of America

**Keywords:** miR-22, MDC1, c-Fos, differentiation, DNA repair

## Abstract

Terminally differentiated cells have a reduced capacity to repair double-stranded breaks (DSB) in DNA, however, the underlying molecular mechanism remains unclear. Here, we show that miR-22 is upregulated during postmitotic differentiation of human breast MCF-7 cells, hematopoietic HL60 and K562 cells. Increased expression of miR-22 in differentiated cells was associated with decreased expression of MDC1, a protein that plays a key role in the response to DSBs. This downregulation of MDC1 was accompanied by reduced DSB repair, impaired recruitment of the protein to the site of DNA damage following IR. Conversely, inhibiting miR-22 enhanced MDC1 protein levels, recovered MDC1 foci, fully rescued DSB repair in terminally differentiated cells. Moreover, MDC1 levels, IR-induced MDC1 foci, and the efficiency of DSB repair were fully rescued by siRNA-mediated knockdown of c-Fos in differentiated cells. These findings indicate that the c-Fos/miR-22/MDC1 axis plays a relevant role in DNA repair in terminally differentiated cells, which may facilitate our understanding of molecular mechanism underlying the downregulating DNA repair in differentiated cells.

## INTRODUCTION

Terminally differentiated cells are characterized by permanent withdrawal from the cell cycle and a lack of genome replication. Although the majority of cells in multicellular organisms are terminally differentiated and do not proliferate, most studies on the DNA damage response (DDR) have investigated the response of proliferating cells to genotoxic agents. Thus, little information exists on the effects of DNA damage in terminally differentiated cells. While resistance to various genotoxic stressors increases during cellular differentiation, DNA repair is progressively attenuated upon cellular differentiation, and therefore, terminally differentiated cells accumulate numerous chromosomal lesions [1, 2]. However, only some aspects of the molecular mechanisms that cause attenuation of DNA repair in terminally differentiated cells have been elucidated, such as the transcriptional downregulation of specific repair proteins [3] and impairment of functional DDR signaling [4–6].

DSBs are dangerous DNA lesions. If left unrepaired or repaired incorrectly, they may result in a massive loss of genetic information, chromosomal aberrations, or cell death. The eukaryotic DDR system allows cells to sense DNA damage and orchestrate appropriate cell-cycle checkpoints and DNA repair mechanisms [7]. DSBs activate the DDR by triggering the kinase activity of the ataxia telangiectasia mutated protein (ATM), thereby initiating a signaling cascade in which the adaptor protein mediator of DNA damage checkpoint 1 (MDC1) is recruited to DSB sites. MDC1 then amplifies ATM signaling activity and contributes to the recruitment and retention of additional DDR factors at sites of DNA damage [8]. Thus, MDC1 has been termed a master regulator, modulating the specific chromatin microenvironment required to maintain genome stability. Notably, loss of MDC1 impairs DNA repair, compromises genome integrity and enhances cancer susceptibility in mice [9, 10]. Also, a reduction or lack of MDC1 has been observed in breast and lung carcinoma cells in humans [11], an observation that has both clinical and mechanistic implications.

Several studies have also shown that microRNAs (miRNAs) are aberrantly expressed in human cancer tissues and that the modulation of miRNA expression levels may be a feasible therapeutic strategy for cancer [12, 13]. Because miRNAs modulate tumor sensitivity to radiation and anticancer drugs in cancer cells through regulation of DNA repair, identifying miRNAs that contribute to regulation of the DDR is important for the development of therapeutics aimed at modulating the response to radiotherapy or chemotherapy. To date, several miRNAs have been shown to be involved in regulation of the DDR by specifically targeting DNA repair proteins [5, 14–21]. Despite these observations, mechanisms involving miRNAs during DNA repair, especially under pathophysiological conditions, remain mostly undefined.

The purpose of this study was to identify miRNAs that regulate the cellular response to DNA damage in terminally differentiated cells and to determine their function. We examined miRNA expression and found that expression of miR-22 is upregulated in three terminally differentiated cell lines: MCF-7, HL60 and K562 cells. In previous work, we demonstrated that miR-22 regulates DSB repair through downregulaton of MDC1 expression [17]. Here, we have uncovered a new MDC1 regulatory pathway triggered by the c-Fos-dependent upregulation of miR-22 expression during terminal differentiation. Thus, we suggest that the c-Fos/miR-22/MDC1 axis-mediated downregulation of DSB repair induces chromosome instability during terminal differentiation and may represent a therapeutic target for cancer.

## RESULTS

### miR-22 is upregulated in post-mitotic differentiated cells

**Figure 1:**
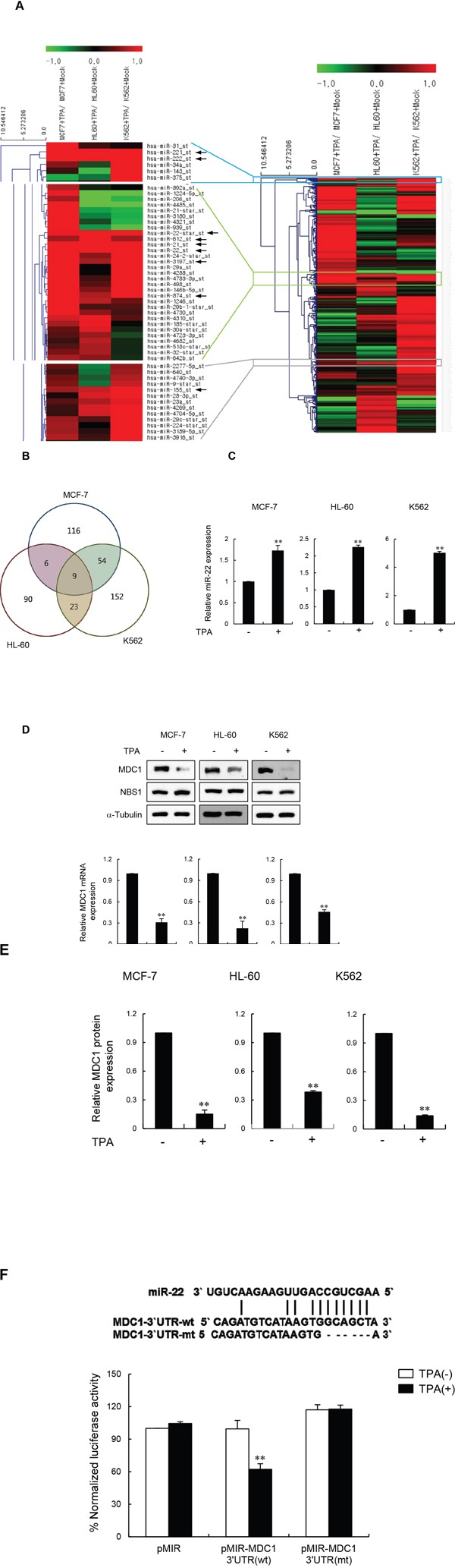
miR-22 downregulates MDC1 expression during terminal differentiation **(A)** Global miRNA expression profile of differentiated human MCF-7, HL60 and K562 cells. The heatmap represents the mean ratio of three independent experiments and shows single-linkage hierarchial clustering, using Pearson squared as a distance metric. miRNA expression in each lane is analyzed relative to expression in control undifferentiated cells. The color bar indicates the expression changes of miRNA: red and green represent upregulation and downregulation, respectively. Arrows indicate upregulated-miRNAs (>1.5 fold) in all three cell lines. **(B)** Venn diagram showing the relationship between the differentially expressed miRNAs from the comparison shown in Supplementary Table 1. **(C)** Quantitative RT-PCR analysis of miR-22 expression in the indicated cells (untreated or treated with TPA). Transcript levels were normalized to U6 expression. **(D)** Western blot (upper panels) and quantitative RT-PCR (lower panels) analysis of TPA-untreated or treated MCF-7, HL60 and K562 cells. RT-PCR signals were normalized to GAPDH expression. **(E)** Quantification of MDC1 Western blot signals from three independent experiments as performed in a using SCION image software (version 4.02 form the Scion Corporation). MDC1 protein levels were normalized using NBS1 and α-tubulin as a loading control. **(F)** Schematic showing the predicted miR-22 binding site in the 3′-UTR of MDC1 mRNA (top). Luciferase assay of indicated cells transfected with MDC1-3′-UTR-wt and MDC1-3′-UTR-mt with or without TPA (bottom). In all panels, mean ± s.d. is shown. **P < 0.01 versus the control group.

To uncover miRNAs that play an important role in the DNA damage response, the human breast cancer cell line (MCF-7) and two human leukemia cell lines (HL60 and K562) were treated with 12-*O*-tetradecanoylphorbol-13-acetate (TPA) to induce terminally differentiated cells. TPA causes MCF-7 cells to become postmitotic differentiated cells [22], and causes HL60 and K562 to terminally differentiate into macrophages and megakaryocytes, respectively. To identify miRNAs that are differentially expressed between undifferentiated and differentiated cells, we used Affymetrix Human Genechip miRNA 3.1 Array (19,724 total mature miRNA probe sets / 1,733 human mature miRNA) using total RNA obtained from the three different cell lines described above, and compared the miRNA expression levels between undifferentiated and differentiated cells by expression fold-change (>1.5-fold) and Student's *t*-test (p<0.01) (Figure 1A and Supplementary Figure 1A-1C). Overall, 116 miRNAs were identified as being differentially regulated between undifferentiated and terminally differentiated MCF-7 cells, 90 miRNAs between HL60 and differentiated macrophages, and 152 miRNAs between the K562 and differentiated megakaryocytes (Figure 1A and Supplementary Table 1). We combined the three data sets into a Venn diagram and observed a total of nine miRNAs that were differentially expressed in all three cell lines (Table 1). Among these, a statistical analysis generated a list of nine miRNAs that were consistently upregulated >1.5 fold in all three terminally differentiated cells, but not in the proliferating cells (Figure 1B, Supplementary Figure 1A-1C and Table 1). Although miR-221 and miR-222 were highly expressed in differentiated cells, they do not target mRNAs for DNA repair. On the other hand, because miR-22 has been described as a differentiation-responsive miRNA [5, 23, 24] and because we have previously reported that it targets MDC1 [17], an important mediator of the DDR [25–27], we were particularly interested in the miR-22.

**Table 1:** Significantly upregulated microRNA in all three cell lines

miRNA	Microarray-MCF-7	Microarray-HL60	Microarray-K562
Hsa-miR-155	1.557	1.724	2.398
Hsa-miR-21	2.928	2.310	1.995
Hsa-miR-22*	1.944	6.466	2.343
Hsa-miR-221	3.017	2.506	226.944
Hsa-miR-222	3.322	2.022	663.603
Hsa-miR-22	2.273	2.910	2.101
Hsa-miR-3197	2.883	1.583	2.862
Hsa-miR-612	1.552	4.916	1.557
Hsa-miR-874	3.885	1.609	2.008

Fold change indicate the expression level of miRNA in TPA-treated cells compared to the untreated cells.

We therefore validated our array data on miR-22 by quantitative RT-PCR. Indeed, miR-22 was consistently upregulated during terminal differentiation of MCF-7, HL60 and K562 cells (Figure 1C). We then investigated MDC1 protein and mRNA expression in MCF-7, HL60 and K562 cells during terminal differentiation. Western blot analysis showed that the protein level of MDC1, but not of other DDR factors such as NBS1, was markedly reduced after TPA-differentiation in all three cell types (Figure 1D, upper panels). Quantification of MDC1 protein from three independent experiments revealed that the level of MDC1, measured after TPA induction, dropped by 6.7-fold in MCF-7 cells, 2.5-fold in HL60 cells, and 8-fold in K562 cells (Figure 1E). In addition, quantitative RT-PCR analysis of the same cells revealed a decrease in MDC1 mRNA levels, indicating that TPA-induced differentiation triggered MDC1 mRNA destabilization (Figure 1D, lower panels). We also treated another differentiation-inducing agent, retinoic acid (RA), with MCF-7, HL60 and K562 cells and measured miR-22 and MDC1 expression. Similar to TPA-treated cells, RA-treated cells also showed increased miR-22 expression and decreased MDC1 expression (Supplementary Figure 2A and 2B).

To test whether MDC1 is directly targeted by miR-22 during terminal differentiation, we tested the effect of miR-22 on luciferase expression from control or full-length MDC1 3′-UTR-containing reporter genes in MCF-7 cells during terminal differentiation. Luciferase activity was reduced more than two-fold by treatment with TPA when compared to control, while deletion of the miR-22 binding site abrogated this effect (Figure 1F). Collectively, these results strongly suggest that the expression of miR-22 is inversely correlated with the expression of MDC1 during cell differentiation and that miR-22 downregulates MDC1 expression at the post-transcriptional level.

### Induction of miR-22 impairs formation of MDC1 foci in differentiated MCF-7 cells

To confirm the differentiation by TPA in MCF-7 cells, we observed cell morphology, lipid accumulation, and the PPARγ expression. MCF-7 cells displayed typical epitheloid characteristics: cells are small and polygonal in shape. After 3 day's exposure to TPA, MCF-7 cells lose their morphology and become rounded and spread out (Supplementary Figure 3A). PPARγ activation induced cells to a more differentiated and caused extensive lipid accumulation in cultured breast cancer cells [28, 29]. Thus, we examined lipid accumulation, and the PPARγ expression. We observed that MCF-7 cells treated with TPA for 3 days accumulate numerous fat droplets in the cytoplasm in comparison with untreated control cells (Supplementary Figure 3B). In addition, PPARγ mRNA increased approximately 2-fold in MCF-7 cells after TPA treatment (Supplementary Figure 3C).

**Figure 2:**
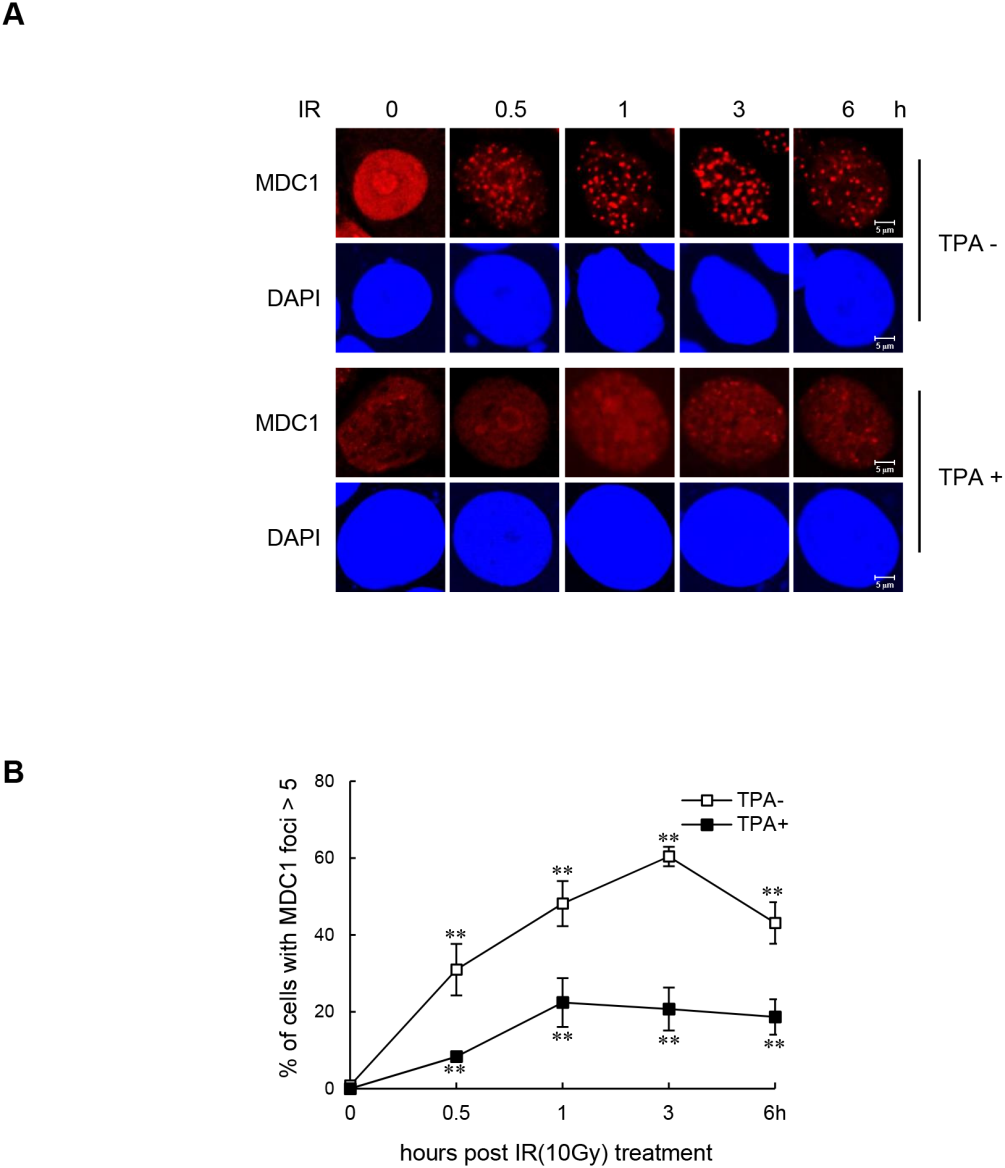
Impaired IR-induced MDC1 foci formation in differentiated MCF-7 cells **(A)** Undifferentiated MCF-7 cells and TPA-differentiated MCF-7 cells were irradiated with 10 Gy and fixed for immunofluorescence staining of MDC1 at the indicated time points. **(B)** Quantification of IR-induced MDC1 foci in undifferentiated MCF-7 cells and TPA-differentiated MCF-7 cells. Results are shown as mean ± s.d. (n = 3); **P < 0.01.

Next, we explored whether reduced expression of MDC1 in differentiated MCF-7 cells is associated with changes in the localization of the protein to DDR-associated foci in the differentiated state. The presence of MDC1 damage foci in response to IR during terminal differentiation was examined by immunofluorescence. When undifferentiated MCF-7 cells were exposed to IR, clearly visible MDC1 foci formed, and the percentage of cells with >5 MDC1 foci continued to increase for three hours post IR-exposure (Figure 2A and 2B). In contrast, cells that were stimulated to differentiate by TPA treatment had little or no MDC1 foci formation after exposure to IR.

**Figure 3:**
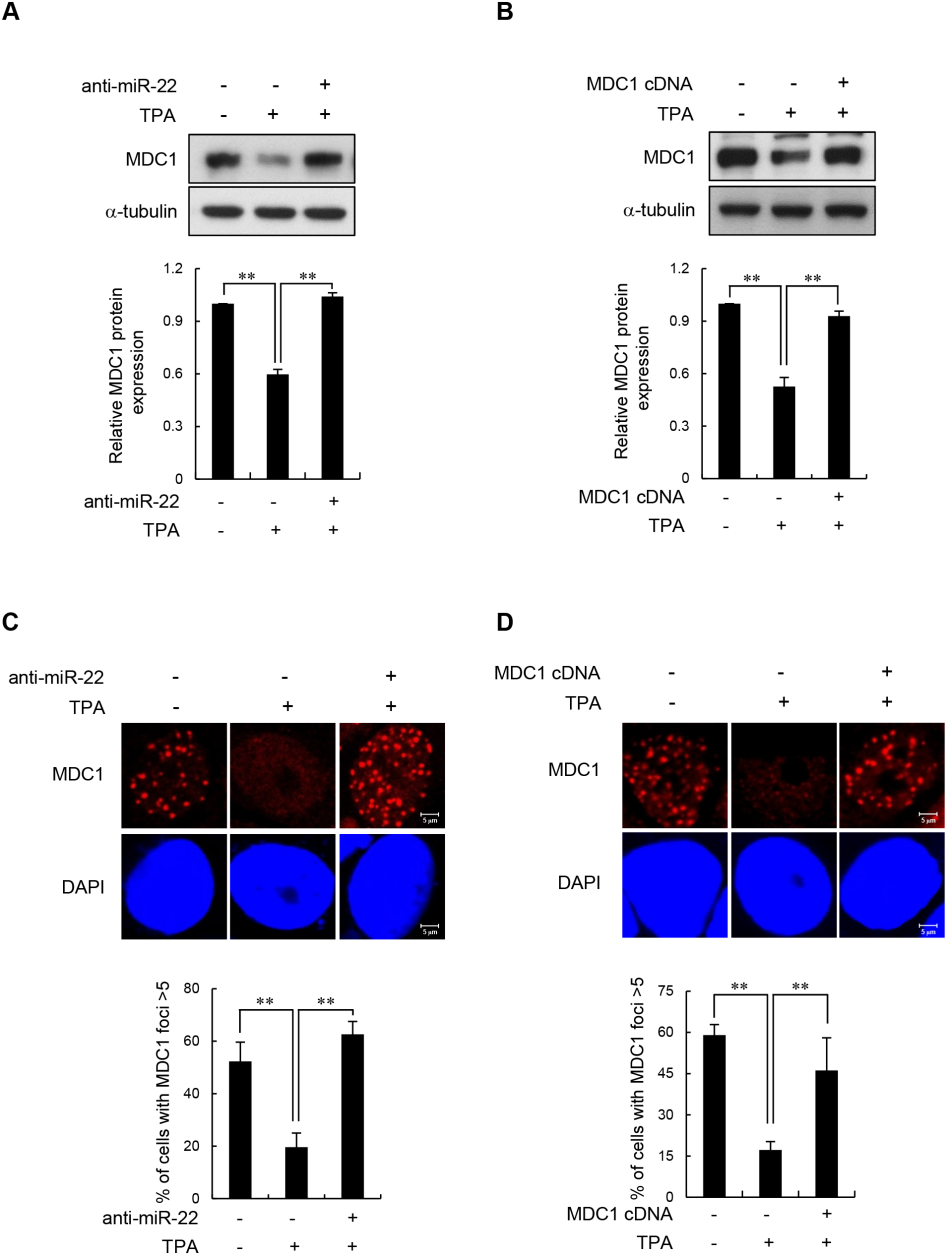
Effect of miR-22 on IR-induced MDC1 foci in differentiated MCF-7 cells (**A** and **B**) Untreated and TPA-treated MCF-7 cells were transfected with anti-miR-22 (**A**) or miR-22-insensitive MDC1 cDNA (**B**). After a 24 h transfection, the cells were irradiated with 10 Gy IR. 3 hr after irradiation, the protein level of MDC1 was measured by Western blot (top). Quantitative densitometry of MDC1 protein expression is shown below, expressed as the mean ± s.d. (n = 3); ** *P* < 0.01. (**C** and **D**) MCF-7 cells underwent the same treatment as A and B and then were analyzed by immunofluorescence for MDC1 foci formation 3 hr postirradiation. DAPI was used for nuclear staining. In all panels, mean ± s.d. is shown; **P < 0.01 versus the control group.

We next showed that TPA-induced differentiation negatively regulates MDC1 expression via miR-22. The effect of TPA-induced differentiation on MDC1 protein expression was effectively reversed by transfecting cells with anti-miR-22 (Figure 3A and Supplementary Figure 4A) and by expressing exogenous MDC1 cDNA that does not harbor the miR-22-binding 3′-UTR (Figure 3B and Supplementary Figure 4B). Consistent with these results, transfection of the anti-miR-22 (Figure 3C) or miR-22-insensitive MDC1 cDNA (Figure 3D) rescued the inhibitory effect of TPA-induced differentiation on MDC1 foci formation as shown by immunofluorescence, suggesting that inhibition of MDC1 foci in TPA-differentiated MCF-7 cells is due to expression of miR-22.

### miR-22 affects DSB repair by modulating MDC1 expression in terminally differentiated MCF-7 cells

**Figure 4:**
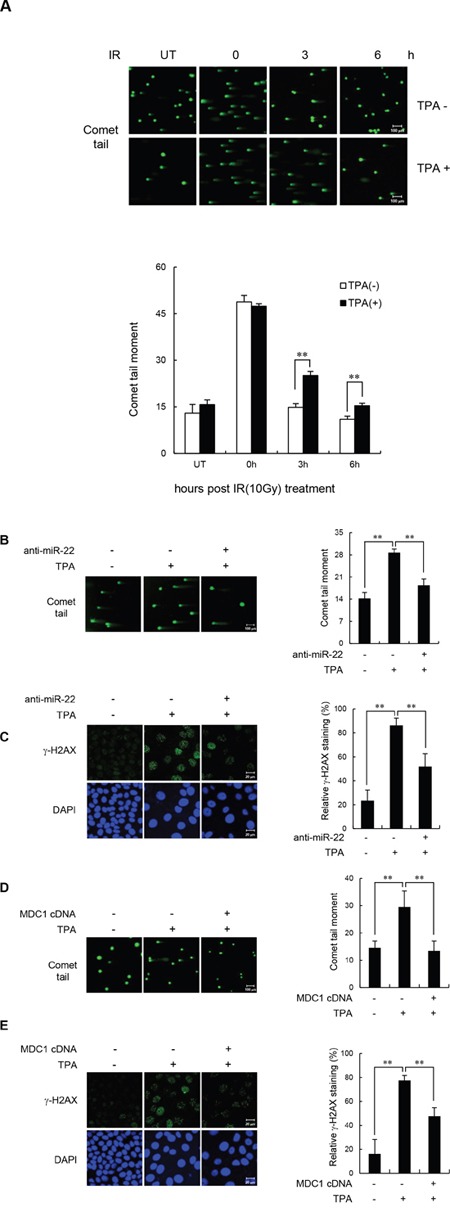
miR-22-mediated downregulation of MDC1 suppresses DSB repair in differentiated MCF-7 cells **(A)** Untreated and TPA-treated MCF-7 cells were irradiated with 10 Gy IR. DSB repair was then measured by a comet assay. (**B** and **C**) Comet assay **(B)** and γ-H2AX staining revealed that transfection of anti-miR-22 rescued DSB repair in TPA-differentiated MCF-7 cells. (**D** and **E**) miR-22-insensitive MDC1 expression decreased comet tail **(D)** and γ-H2AX staining **(E)** and in TPA-differentiated MCF-7 cells. Comet assay and γ-H2AX were measured 3 and 16 hr after irradiation, respectively. In all panels, mean ± s.d. is shown; **P < 0.01 versus control group.

Given the known roles of MDC1 in DSB repair, we investigated whether miR-22-mediated downregulation of MDC1 affects DNA repair in differentiated cells by measuring the persistence of DSBs after IR by single-cell gel electrophoresis (comet assay) as an indicator of unrepaired DSBs. For the comet assay, the undifferentiated and TPA-differentiated MCF-7 cells were irradiated with 10 Gy and collected after incubation for 0, 3, and 6 h. Consistent with a previous report [22], we found that TPA-differentiated MCF-7 cells exhibited extensively longer comet tail “moments” 3 and 6 h after IR treatment compared to undifferentiated cells (Figure 4A). To determine whether the effect of TPA-induced differentiation on DSB repair was mediated by MDC1 and miR-22, we transfected MCF-7 cells with either anti-miR-22 or the miR-22-insensitive MDC1 expression plasmid, and then measured comet tail moments and the levels of γ-H2AX foci, which act as useful surrogate marker of DNA damage. We observed that the impaired DSB repair capacity in differentiated MCF-7 cells was fully rescued by anti-miR-22 (Figure 4B and 4C) or miR-22-insensitive MDC1 (Figure 4D and 4E). The expression of anti-miR-22 and miR-22-insensitive MDC1 showed no significant increase in γ-H2AX staining or comet moments after IR exposure compared to undifferentiated cells. Together, these results provide evidence that endogenous miR-22 induced in response to cellular differentiation affects DSB repair, and suggests that miR-22-mediated down-regulation of MDC1 plays a role in the genomic instability of terminally differentiated cells.

### c-Fos upregulates miR-22 in differentiated cells

**Figure 5:**
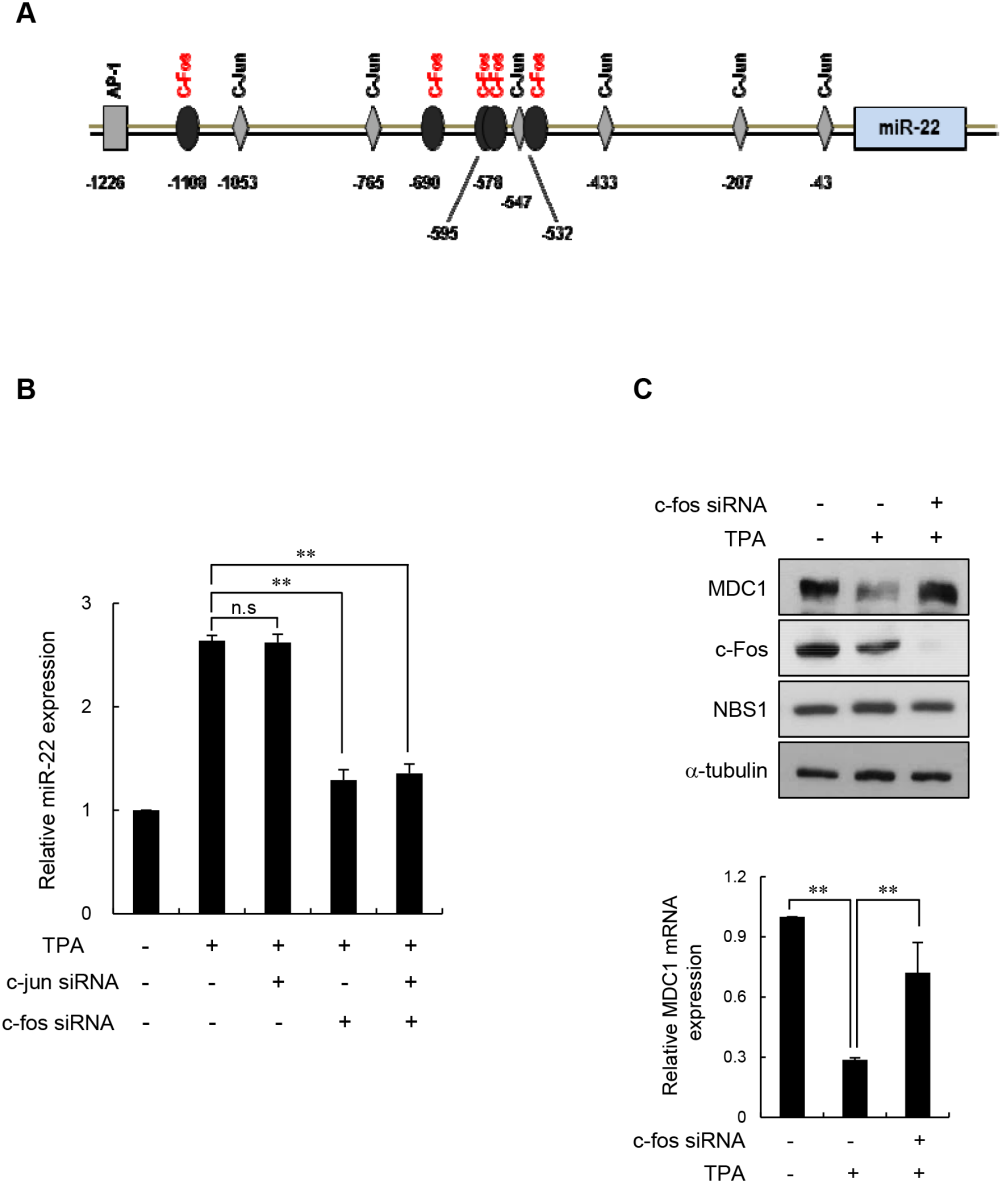
c-Fos negatively regulates MDC1 via miR-22 in differentiated MCF-7 cells **(A)** Representation of the human -1.3 kb miR-22 promoter fragment. Transcription factor analysis of the proximal promoter showing the six c-Jun and five c-Fos binding sites using the PROMO program. **(B)** TPA-differentiated MCF-7 cells were transiently transfected with either c-Jun or c-Fos siRNA. Endogenous miR-22 levels in the indicated cells were measured using real-time qPCR. Data were normalized to U6. Results are shown as mean ± s.d. (n = 3); **P < 0.01. **(C)** TPA-treated MCF-7 cells were transiently transfected with c-Fos siRNA or a control siRNA, and indicated protein levels were determined by Western blotting (top). Expression of MDC1 mRNA was quantitated using real-time qPCR. Data represent mean ± s.d. (n = 3); ** *P* < 0.01.

We then asked how miR-22 expression is regulated during cell differentiation. Expression of miRNAs can be regulated at the transcriptional level between normal and pathological conditions. To determine which transcription factor(s) might influence miR-22 expression in differentiated cells, we performed an in silico analysis of the miR-22 promoter region (1.3 kb upstream of the miR-22 stem loop) using the transcription factor binding site program PROMO [30]. PROMO identified six c-Jun-binding sites at -1053 and -1047, -765 and -759, -547 and -540, -433 and -427, -207 and -200, and -43 and -36 nucleotides, and five c-Fos-binding sites at -1108 and -1099, -690 and -683, -578 and -571, -547 and -540, -532 and -525 nucleotides relative to the miR-22 stem loop (Figure 5A). c-Fos and c-Jun are members of a class of transcription factors that are related to activation protein 1 (AP-1). c-Jun proteins form homodimers or heterodimers with c-Fos proteins through their leucine-zipper domains. The different dimer combinations recognize different sequence elements in the promoters and enhancers of target genes [31]. Of note, c-Fos and c-Jun have been formerly described key transcriptional activators during terminal differentiation [32, 33]. Thus, we hypothesized that c-Fos and/or c-Jun might bind to the core elements of the miR-22 promoter and contribute to upregulation of miR-22 transcription in differentiated cells. To test this, TPA-differentiated MCF-7 cells were transiently transfected with either c-Jun siRNA, c-Fos siRNA, or siRNAs for both c-Jun and c-Fos, and then miR-22 expression levels were measured using quantitative RT-PCR. We observed that knockdown of c-Fos reduced miR-22 levels in TPA-differentiated MCF-7 cells, whereas c-Jun siRNA did not affect miR-22 level in these cells (Figure 5B). Moreover, knockdown of both c-Fos and c-Jun did not further suppress miR-22 expression relative to c-Fos knockdown alone. These data suggest that c-Fos is a key transcriptional regulator of miR-22 in differentiated MCF-7 cells. Most interestingly, transfection of c-Fos siRNA into TPA-differentiated MCF-7 cells restored the MDC1 expression that was suppressed in differentiated cells (Figure 5C). We also observed that the reduction of MDC1 protein expression by retinoic acid was restored by c-fos siRNA (Supplementary Figure 5). These results suggest that that c-Fos stimulates miR-22 expression, which in turn downregulates MDC1 expression during terminal differentiation.

### c-Fos depletion led to a decrease in MDC1 damage foci and DSB repair in differentiated MCF-7 cells

**Figure 6:**
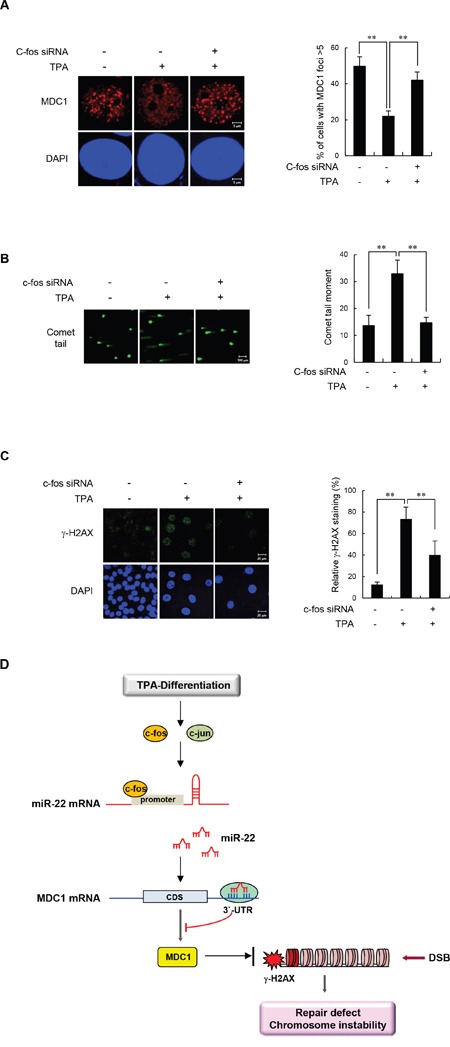
c-Fos knockdown increases IR-induced MDC1 foci and improves DSB repair in differentiated MCF-7 cells **(A)** Untreated or TPA-differentiated MCF-7 cells were transfected with control or c-Fos siRNA and irradiated with 10 Gy of IR. Cells were then fixed for immunofluorescent staining for MDC1 3 hours after IR. Nuclei were counterstained with DAPI. The representative images of MDC1 immunostaining are shown in the left panels. Quantification of relative percentage of cells with at least 5 foci is shown in the right panels. **(B, C)** Untreated or TPA-differentiated MCF-7 cells were mock transfected, transfected with control or c-Fos siRNA and irradiated with 10 Gy of IR. Cells were then analyzed by comet assay 3 hours after IR **(B)** and by γ-H2AX staining 16 hours after IR **(C)**. DAPI was used for nuclear staining. Results are shown as mean s.d. (n = 3); **P < 0.01. **(D)** A model for linear signaling pathway of c-Fos-miR-22-MDC1 axis.

Having observed the effect of c-Fos on MDC1 expression in differentiated cells, we set forth to determine whether c-Fos is critical for DDR function of MDC1 in differentiated cells. To this end, undifferentiated and TPA-differentiated MCF-7 cells were monitored for IR-induced MDC1 foci formation during c-Fos siRNA knockdown. If c-Fos regulates MDC1 function in differentiated cells, then we would predict that IR-induced MDC1 foci formation would increase in parallel with decreased expression of c-Fos. As shown in Figure 6A, transfection of c-Fos siRNA into TPA-differentiated MCF-7 cells completely rescued IR-induced MDC1 foci formation. These data support an operational model in which c-Fos mediates an increase in miR-22 expression that impacts MDC1 function in differentiated cells.

To directly demonstrate the connection between enhanced c-Fos expression and impaired DSB repair in differentiated cells, we transfected TPA-differentiated MCF-7 cells with c-Fos siRNA and measured the persistence of DSBs by comet assay after IR as an indicator of unrepaired damaged DNA, (Figure 6B) and also by γ-H2AX staining (Figure 6C). Importantly, TPA-differentiated MCF-7 cells transfected with control siRNA had significantly higher residual DNA damage relative to undifferentiated cells. Conversely, TPA-differentiated MCF-7 cells transfected with c-Fos siRNA had significantly lower amounts of DSBs. Taken together, these results suggest that induction of c-Fos and the subsequent ability to induce miR-22 expression is responsible for the impaired DDR function of MDC1 in differentiated cells.

## DISCUSSION

Differentiated cells do not engage in genome replication, and most of the genome remains silent. Because terminally differentiated cells are continuously exposed to metabolites and exogenous DNA-damaging insults, DNA repair capacity in these cells is expected to be essential for maintaining genomic integrity. Therefore, clarifying variation in the DNA repair process in differentiated cells may allow us to better understand the relationships among genome integrity, cellular differentiation, and senescence, and may even provide new theoretical paradigms for developing regenerative medicines. Although our knowledge on the efficiency of DNA repair and mode of the DDR signaling pathway along with the cellular differentiation program is still quite limited, it is now generally accepted that terminally differentiated cells have a reduced capacity for DNA repair [1, 2].

Changes in the expression of miRNAs can rapidly and effectively regulate many cellular activities, including DNA repair activity in differentiated cells, and miRNA expression differs between dividing cells and differentiated cells. In a recent study, the miR-24 family was reported to be upregulated in differentiated hematopoietic cells and T cells, and was shown to directly downregulate H2AX expression, thereby inhibiting DSB repair and enhancing chemosensitivity [5]. However, H2AX expression and its function are intact in terminally differentiated astrocytes [4], and we did not observe any significant changes in endogenous H2AX expression levels in terminally differentiated MCF-7 cells, indicating that miR-24 affects H2AX expression depending on the cellular context. We now demonstrate that miR-22 is upregulated in differentiated breast cancer MCF-7 cells as well as in the human leukemic cell lines HL60 differentiated into macrophages and K562 cells differentiated into megakaryocytes. Recent miRNA studies have also provided compelling evidence for an upregulation of miR-22 in various postmitotic cells [5, 23, 24]. We have recently discovered that upregulation of miR-22 leads to a downregulation of MDC1, consequently suppressing efficient DSB repair [17]. MDC1 plays a central role in the DDR by orchestrating DSB repair and checkpoint activation [8]. DSBs are dangerous DNA lesions that cause mutations and genomic rearrangements and consequently contribute to tumorigenesis. DSB repair is therefore an essential way for cells to maintain genome stability and to prevent cancer [7, 34, 35]. Thus, either malfunction or loss of MDC1 is directly linked to genomic instability. Intriguingly, MDC1 expression is also suppressed in terminally differentiated astrocytes before and after exposure to IR [4]. In view of these data, we hypothesized that the down-regulation of MDC1 by miR-22 could be a key factor impairing DSB repair in differentiated cells.

In the present study, we have shown that the levels of MDC1 protein and mRNA were significantly reduced in several terminally differentiated breast and hematopoietic cell lines. To demonstrate that miR-22 effectively targets and downregulates MDC1 in terminally differentiated cells, we inhibited miR-22 during terminal differentiation and monitored MDC1 localization and the DDR function of MDC1. Indeed, miR-22 knockdown completely restored the level of MDC1 expression as well as the recruitment of MDC1 to the DBS sites in terminally differentiated MCF-7 cells. Thus, there is a possibility that attenuated DNA repair in differentiated cells may be dependent on miR-22. Of note, the inhibition of miR-22 in TPA-differentiated MCF-7 cells significantly decreased levels of DNA damage, which indicated an inverse correlation between the level of miR-22 expression and cellular DNA repair capacity in terminally differentiated cells. Terminally differentiated MCF-7 cells expressing high levels of miR-22 exhibited a DSB repair defect, whereas knockdown of miR-22 completely rescued DSB repair in these cells, further indicating that miR-22 regulates DNA repair through MDC1 during terminal differentiation. To provide further evidence of a connection between downregulation of MDC1 and the reduction of DNA repair during terminal differentiation, we overexpressed miR-22-insensitive MDC1 in TPA-differentiated MCF-7 cells and measured DSB repair. Our results showed that overexpression of MDC1 completely restored the function of MDC1 in the DDR and DSB repair in terminally differentiated cells. These results suggest that miR-22 plays an important role in the DDR via regulation of MDC1 expression in differentiated cells.

Differentiation-inducing agents, such as retinoic acid, are widely used for clinical cancer therapy and chemoprevention as differentiation therapy of human cancer [36–38]. Relapsed or refractory cancer that is resistant to differentiation-inducing agent is a clinically significant problem. Acquired resistance through genetic mutation is a major mechanism for cancer drug resistance and accounts to the short life of targeted therapy in human cancer. Mechanically, however, very little is understood about how resistant mutations are actually acquired during cancer therapy. Thus, identifying molecular mechanisms that regulate DNA repair activity in terminal differentiated cancer cells is crucial for cancer prevention and treatment, and the development of cancer sensitization therapy after chemotherapy or radiation therapy. In the present study, we have established that miR-22 is upregulated by the transcription factor c-Fos during terminal differentiation, establishing a linear signaling pathway: differentiation → c-Fos → miR-22 → MDC1. We demonstrated that c-Fos upregulates miR-22 expression in differentiated cells via direct binding to the miR-22 promoter, leading to downregulation of MDC1 and reduction in DSB repair, suggesting that c-Fos induces accumulated DSBs and prevents their repair, at least to some extent, through downregulation of MDC1 (Figure 6D). Thus, our findings suggest that c-Fos/miR-22/MDC1 might act as a sensitizer in cancer therapy and accompany anticancer drug or radiation therapy to enhance therapeutic efficacy and to improve the chance recovery from cancer.

Taken together, our results provide strong evidence that c-Fos dependent upregulation of miR-22 has an important role in regulating DNA repair in differentiated cells by repressing the DDR function of MDC1, promoting accumulation of DNA damage and an environment for genomic rearrangement. Elucidation of this mechanism offers opportunities for applications targeting miR-22 in therapeutic interventions.

## MATERIALS AND METHODS

### Cell culture and TPA treatment

The human breast cancer MCF-7 cells, the human promyelocytic leukemia HL-60 cells and the human erythroleukemic K562 cells were obtained from the American Type Culture Collection (ATCC, Rockville, MD, USA) were cultured in RPMI 1640 medium (Invitrogen, Carlsbad, CA, USA), supplemented with 10% heat-inactivated fetal bovine serum (FBS; Biowest, Riverside, MO, USA), 100 units/ml penicillin, and 100 μg/ml streptomycin sulfate (Invitrogen, Carlsbad, CA, USA). All cells were maintained in a humidified incubator containing 5% CO_2_ at 37°C. To induce DNA double strand breaks, exponentially growing cells were irradiated from ^137^Cs source (Gamma cell 3000 Elan irradiator, Best Theratronics, Ottawa, Canada) at different doses depending on the types of experiments and allowed to recover at 37°C. For terminal differentiation, MCF-7 cells were treated with 12-O-tetradecanoylphorbol-13-acetate (TPA: Sigma), final concentration of 100 nM for 3 days. To induce macrophage and megakaryotes differentiation, HL-60 or K562 cells were treated with 32 nM TPA or 16 nM TPA for 2 days, respectively. Another differentiation reagent, Retinoic Acids (RA, Sigma) was treated to induce differentiation. MCF-7 cells were treated with 10 μM RA for 3 days, and HL60 or K562 cells were treated with 0.1 μM for 3 days or 3 μM for 4 days, respectively.

### MicroRNA microarray

Total RNA extracted from untreated and TPA-treated cells and subjected to microarray analysis using Agilent GeneChip miRNA 3.1 array (Affymetrix, Santa Clara, CA, USA) following the Agilent one color miRNA labeling and hybridization kit protocol. The resulting data were processed and analyzed using Affymetrix^®^ Expression Console™ software (Affymatrix). A *p* value of < 0.05 was used to generate lists of differentially expressed miRNA. MicroRNA microarray analysis was performed by e-biogen company.

### RNA isolation and reverse transcription-quantitative real-time PCR (RT-qPCR)

Total RNA was extracted using TRIzol reagent (Invitrogen). For quantitation of MDC1 mRNA, cDNA was synthesized using 1 μg total RNA, random hexamer and M-MLV reverse transcriptase (Invitrogen). Real-time PCR analysis was performed using the SYBR green-based fluorescent method (SYBR premix Ex Taq kit, TaKaRa Bio, Mountain View, CA, USA) and the MX3000P® qRT-PCR system (Stratagene, La Jolla, CA, USA) with specific primers. Primers used for real-time PCR are as follows: *mdc1* forward, 5′-tgctcttcacaggagtggtg-3′ and *mdc1* reverse, 5′-gggcacacaggaacttgact-3′. *ppar-γ* forward, 5′-cttgcagtggggatgt-3′ and *ppar-γ* reverse, 5′-ctttggtcagcgggaa-3′. To quantify miR-22, cDNA was synthesized using Mir-X™ miRNA first-strand synthesis and SYBR qRT-PCR kit (Clontech) according to the manufacturer's instructions. Hsa-miR-22-MIMAT0000077 was used as primer for real-time qPCR. The quantity of transcripts was calculated based on the threshold cycle (C_t_) using the delta-delta C_t_ method that measures the relative of a target RNA between two samples by comparing them to a normalization control RNA (gapdh for mdc1 or U6 for miR-22).

### MicroRNA luciferase reporter assay

Wild type segments of the 3′UTR of MDC1 containing putative miR-22 binding sites and deletion mutants of predicted miR-22 binding sites were cloned into pMIR-REPORT *firefly* luciferase vector (Applied Biosystems) as described previously [17]. For the luciferase activity assay, pMIR-REPORT luciferase vectors containing wild type or mutant 3′UTRs of DNA-PKcs and pRL-TK vector containing *Renilla* luciferase as a transfection control were co-transfected into MCF-7 cells using Lipofectamine 2000 (Invitrogen), and subsequently, the same cells were treated with 100 nM TPA. After 3 days, the luciferase assay was performed using the dual luciferase reporter assay system (Promega, Fitchburg, WI, USA) according to the manufacturer's instructions. Luciferase activity was quantified using a luminometer (Glomax, Promega). The luciferase activity data were normalized to the *Renilla* value, and the results were represented as the average and standard deviation (SD) from triplicate of experiments.

### Anti-miRNA, siRNA and plasmid transfection

For rescue experiments of differentiation, anti-miR-22 (miR-22 antisense-oligonucleotide (ASO), Panagene) and the pcRNA-HA-MDC1 construct were used. Cells were transfected with 50 nM anti-miR-22 or 1 μg of pcDNA-HA MDC1 using lipofectamine 2000 reagent (Invitrogen) according to the manufacturer's instructions, and then same cells were treated with 100 nM TPA for 3 days. To analyze miR-22 promoter, MCF-7 cells were transiently transfected with c-jun siRNA, c-fos siRNA or both c-jun and c-fos siRNA using lipofectamine RNAiMax (Invitrogen), and subsequently, cells were induced differentiation by treatment of TPA. The siRNA target sequences were as follows: c-Jun siRNA, 5’-CGCAGCAGUUGCAAACAUUdTdT-3’: c-Fos siRNA, 5’-AGGAGAAUCCGAAGGGAAAdTdT-3’: Negative control siRNA (Bioneer, Korea), 5’-CCUACGCCACCAAUUUCGUdTdT-3’.

### Western-blot analysis

Cells were lysed in ice-cold RIPA lysis buffer: 50 mM Tris (pH 8.0) containing 150 mM sodium chloride, 1.0% NP-40 (or Triton X-100), 0.5% sodium deoxycholate, 0.1% SDS (sodium dodecyl sulphate), 2 mM EDTA, and protease inhibitor cocktail (Roche, Basel, Switzerland). Equal amounts of proteins were then resolved on 6–15% SDS-PAGE gels, followed by electrotransfer to polyvinylidene difluoride membranes (Millipore, Bedford, MA, USA). The membranes were blocked for 1h in TBST [10 mM Tris–HCl (pH 7.4), 150 mM NaCl, 0.1% Tween 20] containing 5% skim milk at room temperature and then incubated with the indicated primary antibodies overnight at 4°C. Membranes are washed and incubated with appropriate secondary antibodies for 2 h at room temperature and membranes are developed using enhanced chemi-luminescence detection system. The amounts of MDC1 protein were quantified using Scion Image software (Scion Corp.). The following antibodies were used in this study: anti-MDC1 polyclonal antibody [17], anti-NBS1 monoclonal antibody (BD Biosciences, San Jose, CA, USA), anti-c-fos polyclonal antibody (Santa Cruz) and anti-α-Tubulin monoclonal antibody (Santa Cruz).

### Immunofluorescence cell staining

To visualize γ-ray-induced damage foci, cells cultured on coverslips were washed twice with PBS and fixed in 100% ice cold methanol for 10 min, followed by permeabilization with 0.3% Triton X-100 for 15 min at room temperature. Next, the coverslips were washed three times with PBS, followed by blocking with 0.1% bovine serum albumin in PBS for 1 h at room temperature. The cells were immunostained using primary antibodies and the appropriate secondary antibodies conjugated with Alexa Fluor 488- or Alexa Fluor 594 (green and red fluorescence, respectively; Molecular Probes, Eugene, OR, USA). The coverslips were mounted onto the slides using Vectashield mounting medium containing 4′, 6-diamidino-2-phenylindole (DAPI; Vector Laboratories, Burlingame, CA, USA). Fluorescence images were taken under a confocal microscope (Zeiss LSM 510 Meta; Carl Zeiss, Jena, Germany) and analyzed with Zeiss microscope image software ZEN (Carl Zeiss). Percentage was calculated among at least 100 cells by dividing the number of γ-H2AX foci or MDC1 foci-positive cells by the number of DAPI-stained cells.

### Single-cell gel-electrophoresis (comet assay)

Double strand brake (DSB) repair was visualized by neutral single-cell agarose-gel electrophoresis. Briefly, indicated cells were harvested (~10^5^ cells per pellet), mixed with low melting agarose, and layered onto agarose-coated glass slides. The slides were maintained in the dark for all of the remaining steps. Slides were submerged in lysis solution (Cat.#4250-050-01,TREVIGEN® Instructions, Gaithersburg, MD, USA) for 1 h and incubated for 30 min in neutral electrophoresis solution (100 mM Tris, 300 mM Sodium Acetate at pH=9.0). After incubation slides were electrophoresed (~30 min at 1 V/cm tank length), and then gently immerse slide in DNA Precipitation Solution (1 M NH_4_Ac) for 30 min at room temperature. After air-dried, comet slide stained with SYBR green. Average comet tail moment was scored for 40–50 cells/slide using a computerized image analysis system (Komet 5.5; Andor Technology, South Windsor, CT, USA).

### Oil Red O staining

Differentiation of MCF-7 was monitored by measuring cellular lipid accumulation using Oil Red O staining. Differentiated MCF-7 cells in 12-well plates were washed twice with phosphate-buffered saline (PBS), fixed with 4% paraformaldehyde at room temperature for 1 h, washed with 60% isopropanol, completely dried, and then stained with Oil Red O solution (0.5% Oil Red O in isopropanol/water (6:4), Sigma) for 1 h. Stained cells were photographed using Nikon inverted microscope (Nikon, Tokyo, Japan).

### Statistical analyses

Unpaired Student's t-test was used to test for Statistical comparisons of data. The Data are presented as the mean ± SD. *P* value < 0.01 was considered to be statistically significant. GraphPad Prism (GraphPad Software, USA) and Excel (Microsoft) were used for the analyses.

## SUPPLEMENTARY MATERIALS FIGURES AND TABLES




