# Supplementary material for: c-Fos-dependent miR-22 targets MDC1 and regulates DNA repair in terminally differentiated cells
Source: Oncotarget. 2017 Jun 7;8(29):48204–21. doi: 10.18632/oncotarget.18389 (PMC5564639; doi:10.18632/oncotarget.18389)
Supplement: Supplementary file 1 [file oncotarget-08-48204-s001.pdf]

c-Fos-dependent miR-22 targets MDC1 and regulates DNA repair in terminally differentiated cells

SUPPLEMENTARY MATERIALS

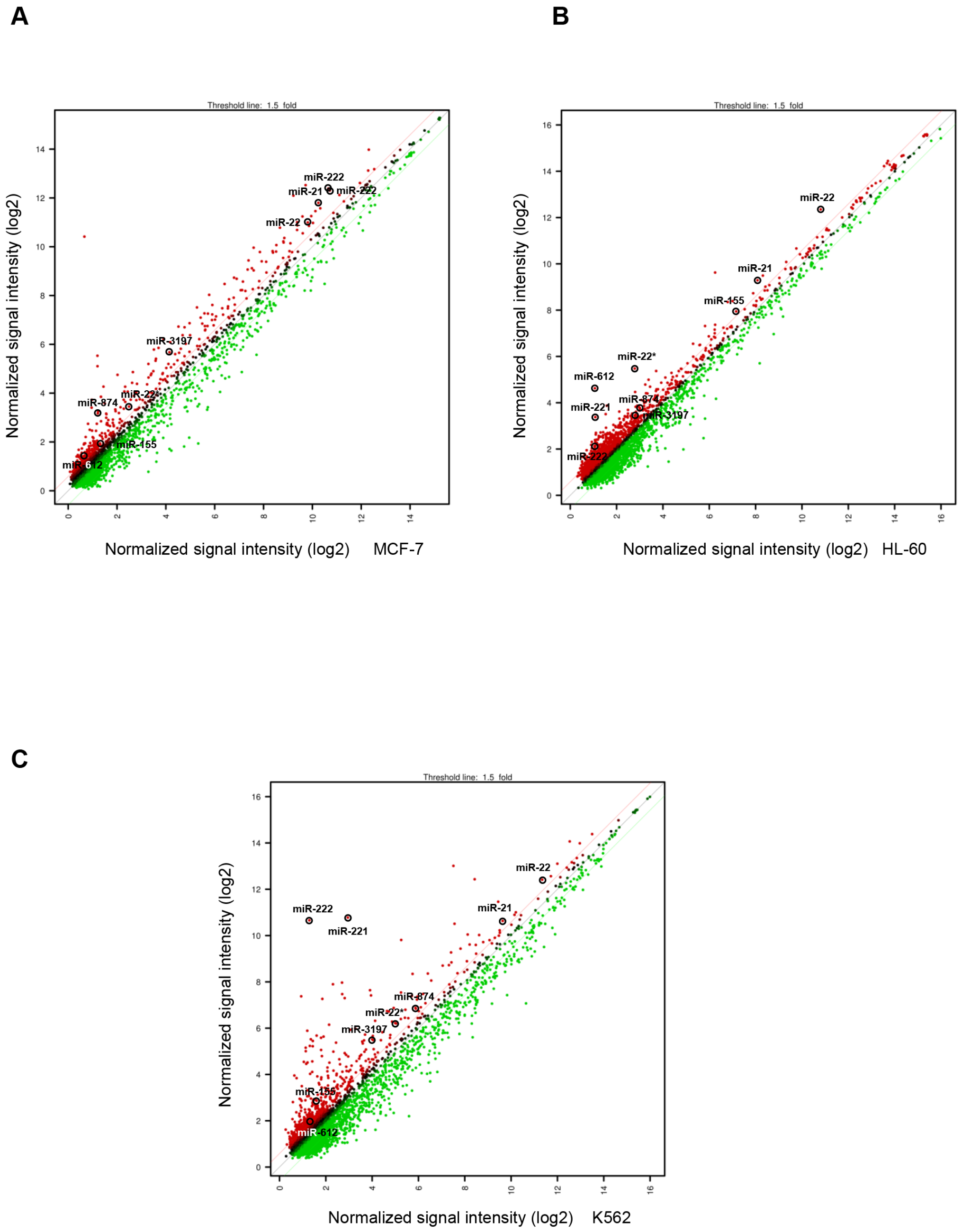

Supplementary Figure 1: Scatter plot of differentially expressed microRNA in the undifferentiated and differentiated cells. **(A-C)** Log-log plot of the expression level of microRNA in undifferentiated cells vs TPA-differentiated MCF-7 **(A)**, HL60 **(B)** or K562 cells **(C)**. Each spot represents an individual microRNA and data are log_2 normalized. Red and Green sopts indicate up- or down regulated microRNA, respectively. Red and Green solid lines show the 1.5 fold change cutoffs. Some microRNAs with highlighted arrows in Figure 1A are marked in this figure.

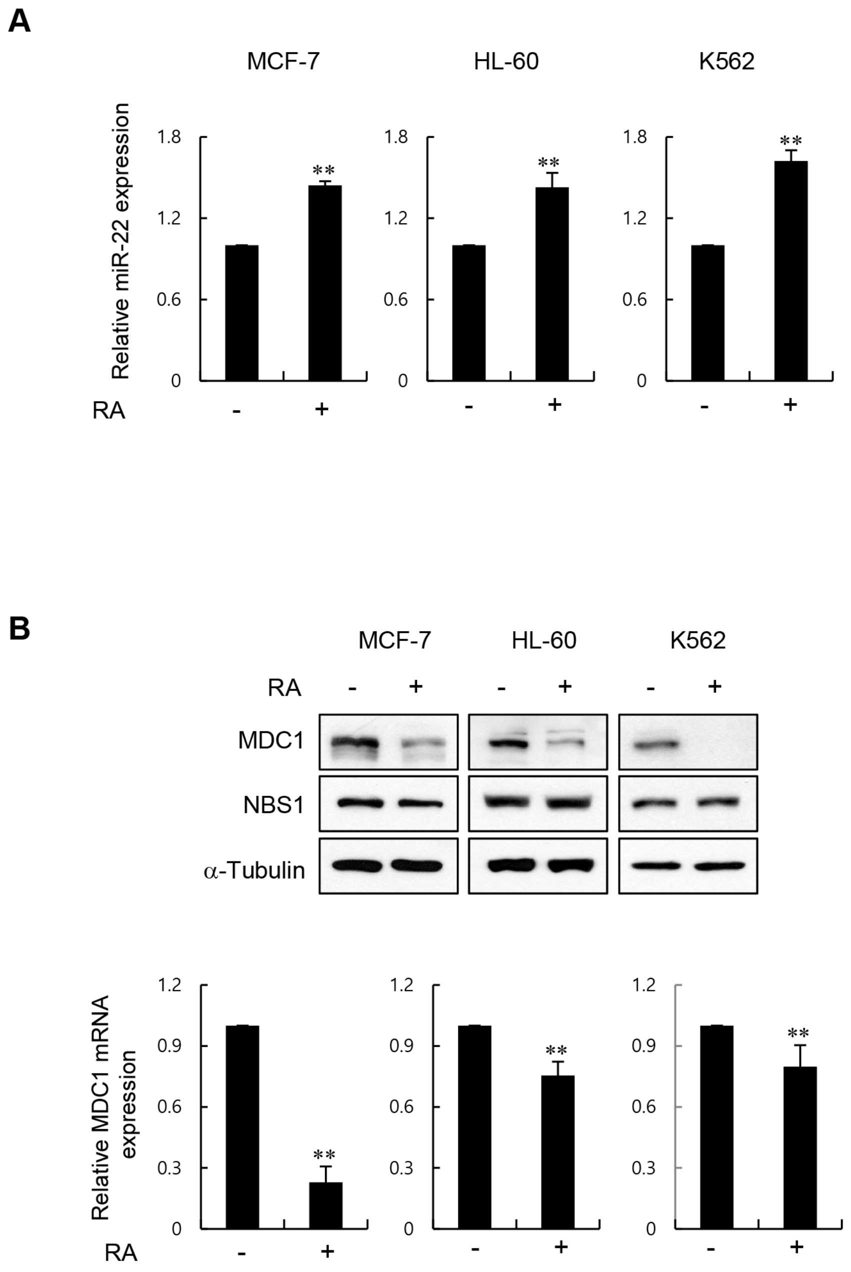

Supplementary Figure 2: Expression of miR-22 and MDC1 in RA-induced differentiated cells. **(A)** Quantitative RT-PCR analysis of miR-22 expression in the indicated cells (untreated or treated with RA). Transcript levels were normalized to U6 expression. **(B)** Western blot (upper panels) and quantitative RT-PCR (lower panels) analysis of RA-untreated or treated MCF-7, HL60 and K562 cells. RT-PCR signals were normalized to GAPDH expression.

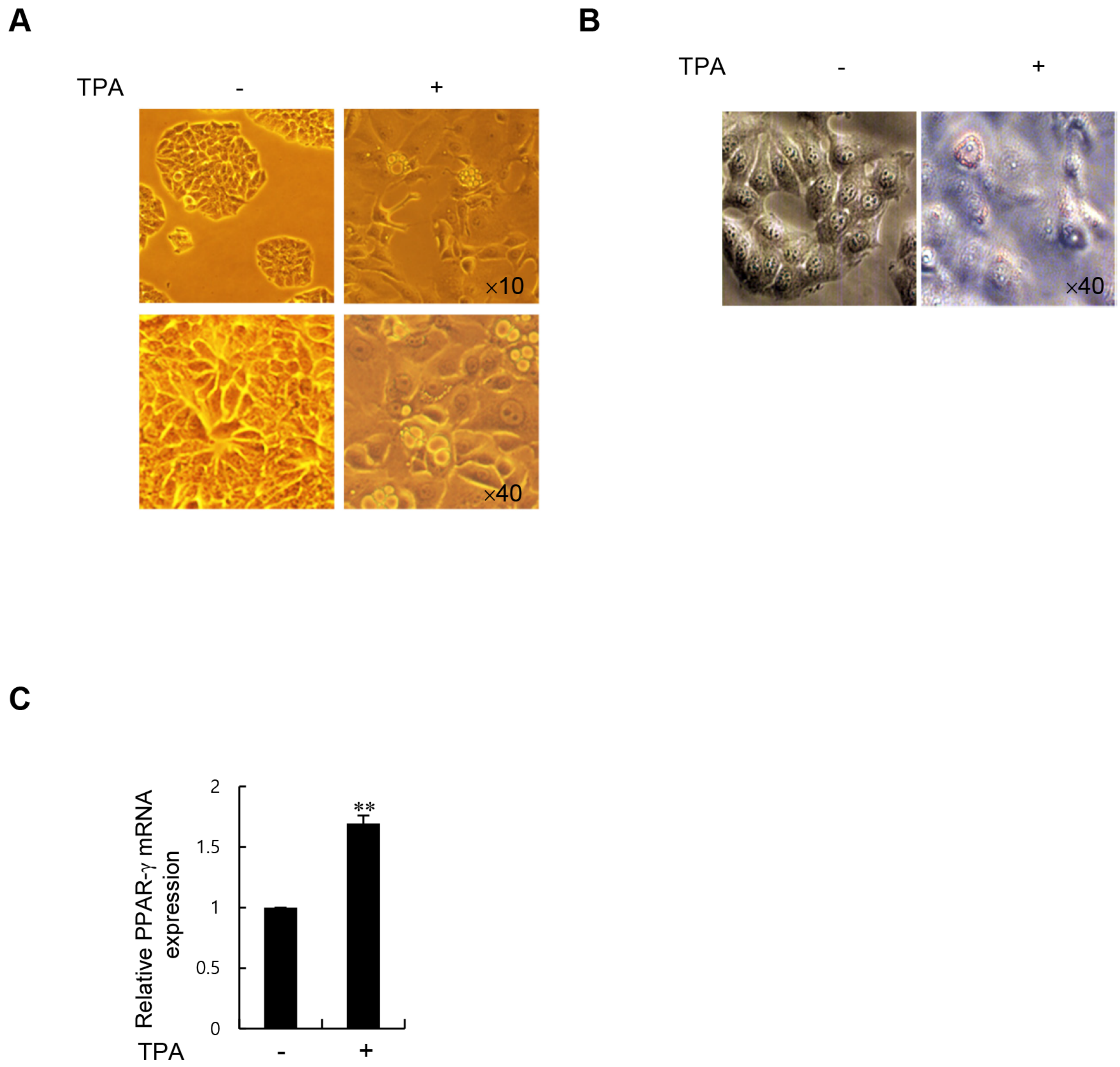

Supplementary Figure 3: TPA induces morphological and biochemical differentiatyion. (A) MCF-7 cell were cultures with or without TPA (100nM) for 3 days. Morphological changes were evaluated by light microscopy using H&E staining. Data are representative of three independent experiments. **(B)** MCF-7 cells were incubated with TPA for 3 days and lipid accumulation was determined using Oil Red O staining. Images were acquired by confocal microscopy. Each was confirmed with three differen preparations. **(C)** Relative PPARγ mRNA expression in MCF-7 cells induced to deffientiate by RA. RT-PCR signals were normalized to GAPDH expression.

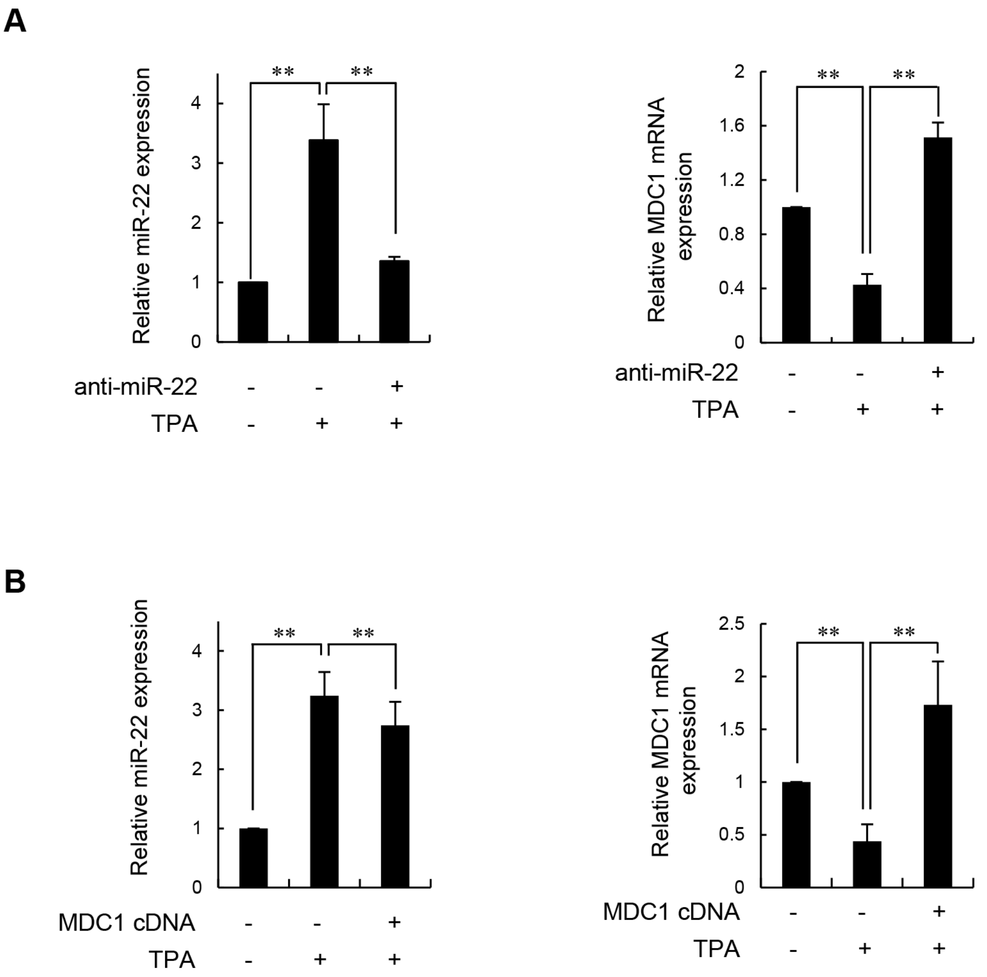

Supplementary Figure 4: Expression of miR-22 and MDC1 mRNA in indicated differentiated MCF-7 cells. (**A** and **B**) Untreated or TPA-treated MCF-7 cells were transfected with anti-miR-22 **(A)** or miR-22-insensitive MDC1 cDNA **(B)**. Two days after the transfection, the levels of miR-22 (left) and MDC1 mRNA (right) were measured by real-time qPCR. Data represent the mean ± s.d. (n = 3); $^{**}P < 0.01$.

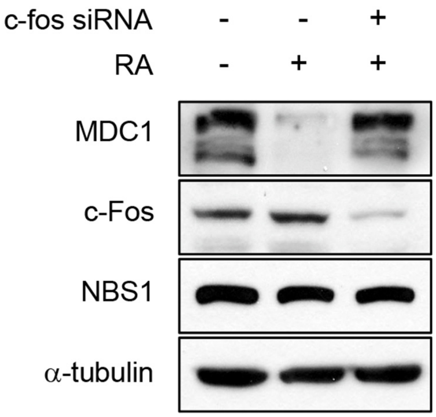

Supplementary Figure 5: c-Fos negatively regulates MDC1 via miR-22 in RA-induced differentiated MCF-7 cells. RA-treated MCF-7 cells were transiently transfected with c-Fos siRNA or a control siRNA, and indicated protein levels were determined by Western blotting.

Supplementary Table 1: Lists of common upregulated miRNAs in all three terminally differentiated MCF-7, HL-60 and K562 cells

See Supplementary File 1
